# Pre-formed DSA and kidney allograft outcomes

**DOI:** 10.1590/2175-8239-JBN-2020-0043

**Published:** 2020-06-01

**Authors:** Melissa Y. Yeung

**Affiliations:** 1 Brigham and Women’s Hospital, Department of Medicine, Boston, MA, USA.; 2 Harvard Medical School, Boston, MA, USA.

Donor-specific antibodies (DSAs) directed against HLA antigens are central to the development of antibody-mediated rejection and chronic allograft vasculopathy, the leading causes of long-term transplant failure. DSAs can be preexisting (preformed) or arise *de novo (dnDSA)* following transplantation. Preformed DSAs occur through previous exposure to alloantigens after blood transfusion, pregnancy or prior transplant. Because preformed DSAs significantly increase the risk of antibody-mediated rejection (ABMR), organs expressing the target antigens of the DSAs are avoided if possible. Using solid-phase, single antigen bead assays, we are now able to identify anti-HLA antibody specificity with high precision and sensitivity. Although there are compelling data for kidney transplants that preexisting DSA is associated with increased risk of rejection, it remains controversial whether antibodies that are detected exclusively by solid-phase assays, and not detected by crossmatch assays, influence long-term graft outcome^1^^,^ 2. 

DSA detected by the complement-dependent cytotoxicity (CDC) crossmatch are considered an absolute contraindication for transplantation because of their propensity to cause hyperacute rejection^3^, whereas DSA detected by other assays represent varying degrees of immunological risk^4^. Transplant centers currently use results of the single antigen bead assay at a pre-defined, low-threshold cutoff to exclude candidates from being eligible for an organ to which they have a DSA. This practice errs on the side of caution by assuming that any low-level antibody affects graft survival and that avoidance of these antigens will result in a better “matched” kidney for the recipient and improve the likelihood of long-term graft survival. However, this approach also restricts a recipient’s access to organs and may lead to longer wait times, particularly for candidates who are highly sensitized. Sensitized candidates have limited access to transplantation in proportion to how broadly their anti-HLA antibodies react with those of the donor. For these patients, use of an organ against which they have preformed DSA may be inevitable. It is estimated that 25% of patients with cPRA will not receive an offer under the current deceased donor organ allocation system in the United States^5^. For these patients, transplantation is possible only if low-level antibody is crossed, or desensitization strategies pursued. It remains unclear which patients would benefit from earlier transplantation with an HLA-incompatible donor versus remaining on dialysis in hopes of finding a more compatible match. 

In this issue, de Sousa *et al.* evaluated the correlation between preformed DSAs and allograft biopsy findings in a cohort of 54 patients. They found no difference in the incidence of ABMR, renal function or proteinuria in the two-year follow-up period between patients who had pre-formed DSAs compared with those who had non-specific HLA antibodies. The primary indications for performing a biopsy were delayed graft function and graft dysfunction, with only 3 cases performed due to dnDSA or an increase in MFI values of the pre-formed DSA. Surprisingly, analysis of Banff histological scores showed patients in the non-DSA group had statistically higher rates of interstitial inflammation and fibrosis, compared to patients that had pre-formed DSAs. 

This data is in discordance to a much larger study by Gossert *et al.* 6, where they found the presence of DSAs was significantly associated with severe interstitial fibrosis and tubular atrophy (IF/TA) in allograft biopsies performed at 1-year post-transplant (adjusted odds ratio of 1.53, 95% CI 1.16-2.01, p=0.002). IF/TA severity was also associated with higher DSA mean fluorescent intensity (MFI) levels. Interestingly, the association between DSA and IF/TA remained significant even in the absence of traditional histological features of ABMR. The disparate findings are likely due to the current study (de Sousa *et al.*) being underpowered. Additionally, the current study does not address whether the preformed DSAs persisted after transplant. In the study by Gossert *et al.* 6, this appears to be an important distinction, since IF/TA correlated with persistence of circulating DSA. Although a higher percentage of patients with preformed DSA developed severe IF/TA (25.7%), 18.4% of patients who had minimal IF/TA also had preformed DSA with no differences in MFI, class or number of DSAs, suggesting that not all pre-formed DSAs may be pathogenic. 

In both studies, all recipients were screened for DSA and had a negative CDC crossmatch prior to transplant. However, flow-cytometric crossmatching was not performed, rendering it difficult to infer the impact of low-level DSAs. Prior data from a large, multicenter study by Orandi *et al.* 2 showed that increasing degrees of HLA-incompatibility, from DSA detected only by single antigen bead testing to flow crossmatch positivity to CDC crossmatch positivity, were associated with higher rates of graft loss and death. However, graft survival was similar if patients had a negative CDC and negative flow-cytometric crossmatch, regardless of the presence of DSA. In contrast, another large, multicenter study by Ziemann *et al.* 7 reported that the presence of preformed DSA was associated with higher all-cause graft loss, with similar rates seen in patients with low-level DSA (MFI<3000) versus higher-level DSA (MFI >3000). Flow crossmatching was not performed, but would likely be negative in patients with low-level DSA. 

In aggregate, it remains unclear whether all preformed DSAs confer similar risk for ABMR, transplant glomerulopathy, fibrosis, and graft loss. Ideally, the avoidance of all preformed DSAs poses the least risk. However, in circumstances where this is not practical or feasible, clinicians must decide whether to proceed with HLA-incompatible transplantation or recommend that their candidate remains on dialysis until a more compatible offer becomes available.
